# CAN TEMPORARY ARTERY CATHETERIZATION EXTEND LIMITS OF ISCHEMIA TIME FOR MACROREPLANTATION?

**DOI:** 10.1590/1413-785220233105e267476

**Published:** 2023-12-18

**Authors:** RAQUEL BERNARDELLI IAMAGUCHI, GUILHERME MOREIRA DIAS, FERNANDA DO CARMO IWASE, MARCELO ROSA DE REZENDE, RAMES MATTAR

**Affiliations:** 1 Universidade de Sao Paulo, Faculdade de Medicina, Hospital das Clinicas, Instituto de Ortopedia e Traumatologia IOT HCFMUSP, Grupo de Cirurgia da Mao e Microcirurgia Reconstrutiva, Sao Paulo, SP, Brazil

**Keywords:** Amputation, Extremities, Forearm, Microsurgery, Catheterization, Wounds and Injury, Amputação, Extremidades, Antebraço, Microcirurgia, Cateterismo, Ferimentos e Lesões

## Abstract

We observe delayed referrals to appropriate Microsurgery Unit and definitive treatment of traumatic limb amputations. Cases with wrist proximal amputations have a deadline for surgical replantation as these configure life-threatening injuries.

**Objective:**

To analyze patients with traumatic proximal wrist upper limb amputations with prolonged ischemic time who underwent temporary artery catheterization to assess stump viability and results.

**Methods:**

A case-series study including all patients with a proximal wrist upper limb amputation and a cold ischemic time equal to or above six hours from 2017 to 2021.

**Results:**

In total, two surgeons operated eight patients who had experienced forearm amputation injuries. Median ischemia time totaled eight hours. All patients required additional surgeries, most commonly split-thickness skin graft or fixation revision (three patients). This study obtained five successful macroreimplantations. The mean cold ischemia time was longer in the group with successful macroreimplantations (7.4 hours) than of the unsuccessful group (9 hours).

**Conclusion:**

Macroreplantations require immediate referral to microsurgery and, although temporary artery catheterization helps surgical decision making, the technique seems to fail to influence outcomes. ***Level of Evidence IV, Retrospective Case Series.***

## RESUMO

Observa-se um atraso no referenciamento dos casos para o tratamento definitivo das amputações traumáticas de membros no Brasil. Casos com amputações proximais ao punho apresentam um prazo limite para reimplante, sendo lesões que promovem risco de vida ao paciente.

**Objetivo:**

Analisar os macrorreimplantes com tempo de isquemia prolongado submetidos à cateterização temporária da artéria, para determinar a viabilidade do coto de amputação, e seus resultados.

**Métodos:**

Série de casos de todos os pacientes com amputações traumáticas proximais ao punho, cujo tempo de isquemia fria foi igual ou superior a seis horas, entre 2017 e 2021.

**Resultados:**

A amostra foi composta por oito pacientes com amputações traumáticas de antebraço operados por dois cirurgiões. O tempo médio de isquemia foi de oito horas. Todos os pacientes necessitaram de cirurgias adicionais, sendo as mais comuns o enxerto de pele ou a revisão da fixação óssea. Sucesso do macrorreimplante foi observado em cinco pacientes. O tempo médio de isquemia fria foi maior no grupo com sucesso no macrorreimplante (7,4 horas) quando comparado com o grupo sem sucesso (9 horas).

**Conclusão:**

Os macrorreimplantes necessitam de transferência imediata para serviços especializados, e, apesar de a cateterização temporária arterial auxiliar no manejo cirúrgico, a técnica parece não interferir nos resultados. **Nível de Evidência IV, Série de Casos.**

## INTRODUCTION

Upper limb macroreimplants with wrist proximal amputations represent life-threatening injuries that are associated with high-energy trauma. The decision to reimplant the amputated limb should be based on patients’ clinical conditions and amputation stump techniques, according to injury type, amputation level, the conditions of stump soft tissues, and cold or warm ischemic time.

A recurrent problem in health systems refers to the prolonged time between the trauma of the limb and the moment in which the patient is received in the service that will perform such surgical procedure. This referral delay increases the chance of complications in patients undergoing macroreimplantation, such as microanastomosis thrombosis, muscle necrosis with rhabdomyolysis, infections, and others. Although some articles have recommended macroreimplantation up to 12 hours of cold ischemia,^1^ Sabapathy et al. ^(^ ^2^ consider that the critical time of cold ischemia would total eight hours, after which, the authors advise against macroreimplantation. Our referral service for complex orthopedics and traumatology cases often receives wrist proximal amputation cases late, forcing Brazilian microsurgeons to decide to try macroreimplantation in these dramatic cases in young patients. This study aims to critically analyze macroreimplants with prolonged ischemia times that received temporary artery catheterization to determine the viability of these amputation stumps and related clinical results.

## METHODS

Our project was submitted to the Research Ethics Committee under CAAE: 51739221.8.0000.0068. Informed consent forms were obtained from all patients following Resolution 466/12 of the National Research Ethics Commission.

Individuals who were referred for surgical treatment of their traumatic upper limb injuries from 2017 to 2021 were included in this study. Inclusion criteria consisted of:

- Wrist proximal amputations
- Mechanism of injury: avulsion
- Cold ischemia times equal to or greater than six hours
- Patients aged 18 years or above
- The presence of appropriate clinical and technical conditions to macroreimplant limbs

For statistical analysis, SPSS, version 20.0 (SPSS Inc^®^, Chicago, IL, USA), was used for descriptive statistics and univariate analysis via the Student’s *t-*test for quantitative data. In the descriptive analysis, intraoperative technical data (need for venous grafts, vessels used for arterial anastomosis, number of microanastomoses, venous system used for microanastomosis), total ischemic time, complications, and additional surgical procedures were evaluated.

The selected cases were transferred to our service so patients could be evaluated. Limb macroreimplantation was indicated after the adequate stabilization of patients and preparation of the technical conditions for the procedure.

The following sequence was set for surgical reimplantations: patients’ admission to the hospital and clinical stabilization, preparation of blood and blood products, radiographs, and transport of the amputated part, correctly packed in a compartment with a saline solution and covered in ice to maintain its cold ischemia.

The total cold ischemic time until the beginning of the surgical procedure was recorded and temporary artery catheterization with revascularization of the amputated part was performed. Regarding venous returns, the vein of the amputated part was freely bled for up to five minutes with adequate hemodynamic stabilization and consent of the anesthesiologist in the room.

A Zeiss OPMI VARIO S88 microscope and 9.0 or 10.0 nylon suture threads (according to vessel diameter) were used.

Prophylactic low-molecular-weight heparin (to prevent postoperative thrombosis in patients who underwent long surgeries), hydration, and analgesia were postoperatively performed at the beginning of recovery together with the intensive care team of the Hospital.

## RESULTS

This study included eight wrist proximal amputations due to six work accidents, one automobile accident, and one train hit from 2017 to 2021. The first and third authors performed all surgeries in cases meeting our inclusion factors (Table 1).

**Table 1:** Descriptive epidemiological analysis of cases.

Case	Age	Gender	Injury level	Mechanism	Ischemia (beginning of surgery)	Associated injuries
1	30	F	Proximal radius	Avulsion	8 hours	Extensive muscle injury of the amputated arm and forearm
2	37	M	Middle-third forearm	Avulsion	9 hours	Extensive muscle injury in the biceps and brachii muscles Irreparable damage of the ulnar nerve (over 30 cm)
3	24	F	Proximal radius	Avulsion	9 hours	Ipsilateral humerus fracture
4	23	M	Distal forearm	Avulsion	6 hours	Irreparable damage of the ulnar nerve (over 30 cm)
5	27	M	Arm diaphysis	Avulsion	6 hours	Extensive muscle injury of the arm
6	37	M	Distal forearm	Avulsion	7 hours	Amputation of the second finger + open fractures on the first and third fingers of the contralateral hand
7	23	F	Proximal radius	Avulsion with crushing	10 hours	Degloving up to the proximal third of the humerus
8	34	M	Proximal radius	Crushing followed by avulsion	9 hours	Vascular segmental lesion in the proximal third of the forearm and lesion of the palmar arch in the hand

**Figure 1:**
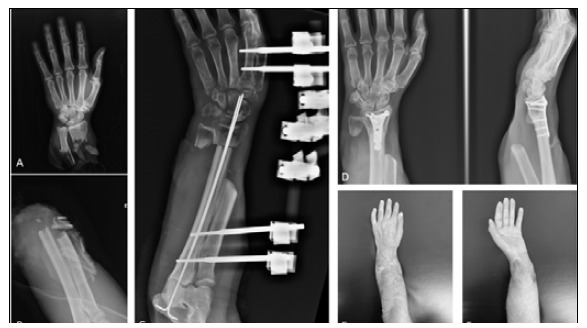
Case 6: (A, B) X-ray of the amputated limb on arrival at the hospital; (C) Radiography after three months of reimplantation showing no bone consolidation; (D) Image after eight years of surgery and a synthesis revision with good consolidation; (E) Clinical image of the limb after eight years.

Patients’ age ranged from 23 to 37 years, averaging 29.4 years. Cold ischemic time ranged from six to 10 hours (standard deviation of 1.5 hours) with a mean of eight hours. The mean time of cold ischemia totaled 7.4 hours (standard deviation = 1.5 hours) for the group with successful macroreimplantations and nine hours (standard deviation = 1.0 hours) for the group with unsuccessful macroreimplantations (no statistically significant difference p = 0.12) (Figure 1).

**Figure 2:**
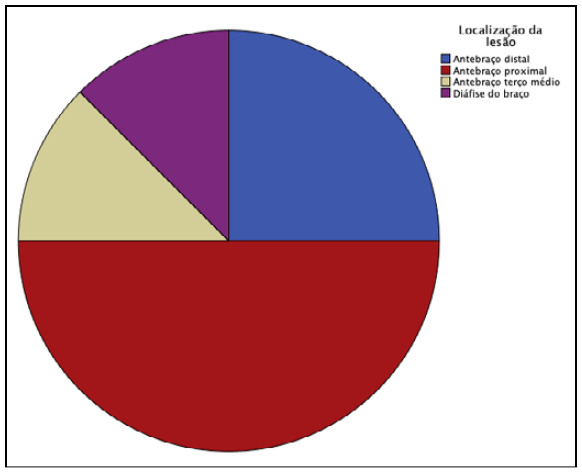
Distribution by amputation level.

**Figure 3:**
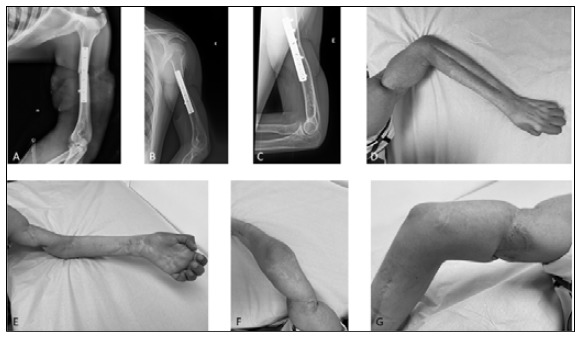
Case 5: (A) Postoperative radiography with synthesis with plate and screws; (B, C) X-ray after nine years of surgery, showing bone healing; (D, E, F, G) Clinical images of the upper limb after nine years.

Cases showed the injury levels and associated injuries in Table 1. The most common associated injury was extensive muscle injury (Figures 2 and 3).

**Figure 4:**
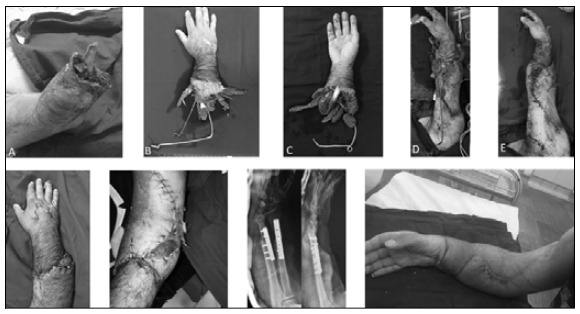
Case 2: (A, B, and C) Upper limb and amputated forearm; (D and E) Intraoperative images; (F and G) Appearance after one week of surgery; (H) Forearm X-ray after conversion for synthesis with a screw plate; (I) Aspect of the upper limb at follow-up.

The team prepared stumps on a sterile operating table with adequate debridement, tendons, and nerves for repair (if feasible), and arteries and veins for microanastomoses. Each case underwent bone shortening and bone fixation preparation as needed (Figure 4).

To reduce the time of additional intraoperative ischemia, all patients received artery catheterization with a silicone catheter before the steps to reimplant the amputated limb to quickly revascularize it. Patients also underwent free vein bleeding for five to 10 minutes to venously drain their stumps, as per the literature. ^(^ ^3^ The team adequately performed water support and volume replacement with blood and hydroelectrolytic products to replace volume due to increased bleeding stemming from temporary arterial catheterization.

**Figure 5:**
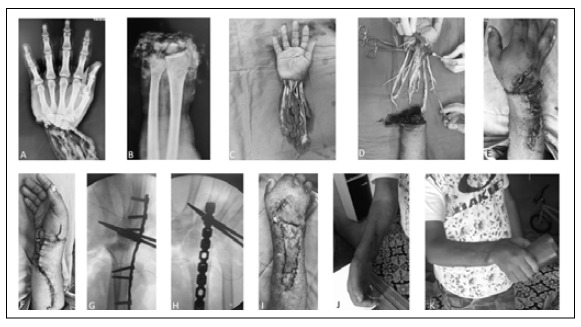
Case 4: (A and B) X-rays of the wrist and amputated hand; (C) Appearance of the hand amputated by avulsion; (D) Debridement of non-viable tissue; (E and F) Final appearance after surgery; (G and H) Radioscopy imaging after wrist arthrodesis; (I) Image after skin graft surgery showing good integration; (J) Clinical image of the upper limb at follow-up; and (K) Patient holding an object.

After revascularizing amputation stumps via temporary catheterization, this study analyzed patients’ clinical stability and the viability of amputation stumps (by attesting to the absence of reperfusion ischemia, which could occur due to prolonged ischemia) and indicated macroreimplantation for the eight evaluated patients. After temporary artery catheterization, the team released muscle compartments, inspected the stumps, and debrided the segments without perfusion or bleeding by observing soft tissues (including the muscles) (Figure 5).

Then, reimplantation followed the conventional steps in the literature. The surgical team performed fixation with plate and screws in five cases; with an external fixator in one case; and with Kirschner wires in one case (due to the absence of suitable material for urgent fixation). Moreover, one patient underwent wrist arthrodesis (Table 2).

**Table 2:** Variables of the operative technique.

Case	Tenorrhaphy or Myorrhaphy	Microneurorrhaphy	Arterial anastomosis	Venous anastomosis
1	Foream flexor and extensor muscle mass	Median and ulnar nerves	Brachial artery	A vena comitans of the brachial artery and a superficial vein
2	Deep flexor tendons of the fingers, long flexor tendon of the thumb, and finger and wrist extensor muscle mass	Median nerve with graft	Proximal ulnar artery and distal radial artery	A vena comitans of the ulnar artery and a superficial vein
3	Foream flexor and extensor muscle mass	Median nerve	Ulnar artery	A vena comitans of the ulnar artery and a superficial vein
4	Superficial and deep flexor tendons of the fingers and finger extensors	Median nerve	Ulnar artery	A vena comitans of the ulnar artery and a superficial vein
5	Myorrhaphy of anterior and posterior muscle bellies	Median nerve	Brachial artery	A vena comitans of the brachial artery and a superficial vein
6	Tenorrhaphy of flexors and extensors with solidarization	Median and ulnar nerves	Ulnar artery	Arteriovenous fistula of the radial artery with reflux in the cephalic vein with a saphenous vein graft
7	Tenorrhaphy of flexors and extensors with solidarity	Median Nerve	Ulnar artery	Arteriovenous fistula of the radial artery with reflux in the cephalic vein
8	No procedure	No procedure	Ulnar artery with saphenous vein graft	Amputation

**Figure 6:**
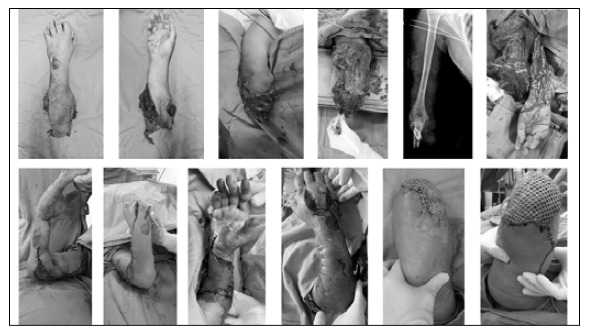
Case 7: Clinical case with the longest cold ischemic time (10 hours). This female patient was hit by a train, which traumatically amputated her right forearm. The case evolved to worsened perfusion four days after macro-reimplantation and the patient chose amputation and regularization of her right upper limb.

All patients required additional surgeries (Table 3) (Figure 6).

**Table 3:** Complications and additional surgeries.

Case	Ischemia (beginning of surgery)	Complications	Additional surgeries
1	8 hours	Muscle necrosis	Serial debridements (three) and amputation of the reimplantation
2	9 hours	Loosened Kirschner wire fixation	Revision two weeks after fixation for open reduction and internal fixation
3	9 hours	Skin necrosis on anastomoses	Anterolateral microsurgical flap of the thigh
4	6 hours	Failure of muscle area coverage.	Skin graft
5	6 hours	Failure of muscle area coverage.	Skin graft
6	7 hours	Pulmonary thromboembolism; Forearm pseudarthrosis	Skin graft and revision of the fixation with consolidation (4 months after surgery)
7	10 hours	Venous congestion and muscle necrosis	Amputation after 5 days
8	9 hours	Lack of intraoperative perfusion	Intraoperative amputation

**Figure 7:**
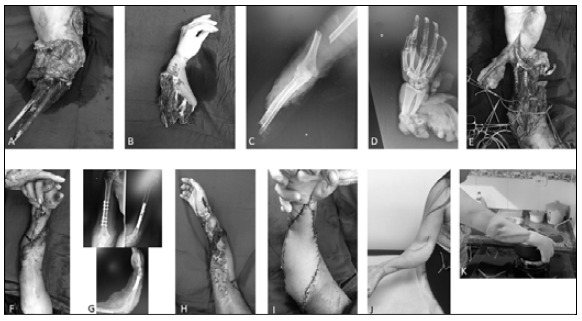
Case 3: (A and B) Upper limb and amputated forearm; (C and D) Radiographs of the upper limb (showing an ipsilateral fracture of the humerus) and amputated forearm; (E) Intraoperative imaging with isolated vessels; (F) Final surgery image; (G) Postoperative X-ray with humerus, radius, and ulna synthesis; (H) Evolution with necrosis of the skin and of the soft portions of the anterior forearm; (I) post-surgery image of the anterolateral flap of the thigh for forearm coverage; (J) Final image of the upper limb; and (K) Evidence of function for activities of daily living.

Of the successful macroreimplantations, five patients reported using their limb functionally, remaining economically active, and working as administrative staff, porter, informal worker, or household worker (Figure 7).

## DISCUSSION

Wrist proximal amputations are rare lesions that require specialized emergency support with clinical patient stabilization and a team specialized in microsurgical surgery. The study of macroreimplantation indications requires the assessment of patients’ history and the characterization of lesions (trauma mechanism, level, elapsed time, and associated injuries) and comorbidities (peripheral arterial disease, diabetes, and smoking cause worse outcomes). In cases of segmental lesion, reimplantation should be rethought in the absence of clinical-hemodynamic stability and prolonged cold or warm ischemia. ^(^ ^4^

The adequate preservation of amputation stumps for macroreimplantation is essential for the best prognosis. Stumps should be wrapped with sterile gauze soaked in a physiological solution or immersed in a saline solution (plain water should be avoided) and placed in a closed compartment surrounded with ice to cool them to about 4°C. ^(^ ^1^ ^), (^ ^4^ In Brazil, delays in patient and stump referrals raise the cooling temperature around the bag holding the stumps to above 4°C, which makes it impossible to determine the adequacy of stump cold ischemia in some cases. In other cases, although extensively described in the medical literature, amputation stumps are place directly on ice, leading to cooling burns and impairing case prognosis.

An available resource in cases with prolonged cold ischemic time (over six to eight hours) is the temporary catheterization of the artery to rapidly revascularize the amputation stump. Nunley, Koman, and Urbaniak^5^ described artery catheterization with or without vein catheterization for venous drainage in 1981, which can be used to evaluate amputation stump viability, especially that of ischemic muscles. However, temporary catheterization is neither a consensus nor should it delay arteriorrhaphies and final venorrhaphies. We recommend its use in cases with prolonged ischemia (over six to eight hours) and vein bleeding from five to 10 minutes with hemodynamic support to eliminate free radicals (including myoglobin, CPK, and potassium) and reduce the risk of acute renal failure or lethal consequences, as per the literature. ^(^ ^3^ ^), (^ ^5^ Chin and Hart^6^ described a case of traumatic wrist amputation, in which they used temporary artery catheterization due to the critical time of warm ischemia (above six hours), gaining time for adequate fixation and other repairs before definitive microanastomosis.

In cases of wrist-proximal upper limb macroreimplantation, the classic sequence of finger reimplantation in the literature should be changed according to ischemic time and surgeons’ preferences. The suggested order for macroreimplantation is:

- Temporary shunt of the artery, according to prolonged ischemic time or surgeon’s preferences (with the advantage of evaluating the viability of the muscle to be debrided)
- Preparation of the amputation stump with aggressive debridement and release of compartments
- Bone shortening and fixation
- Arteriorrhaphy with vascular graft as needed
- Venous anastomoses with vascular graft as needed
- Neurorrhaphy
- Tendon or myotendinous sutures
- Tension-free closure with skin grafts, local flaps, or at a distance as needed.

This surgical technique differs from distal reimplants due to the greater amount of muscle mass in proximal amputations, their greater susceptibility to necrosis due to ischemia, and the need for quicker revascularizations. Although digital amputations can withstand 12 hours of warm ischemia and 24 hours of cold ischemia, macroreimplants tolerate from two to three hours of warm ischemia and six to eight hours of cold ischemia, depending on their level. Unlike Sabapathy et al., ^(^ ^2^ we recommend proximal myotendinous or muscular repair before the closure of soft tissues (rather than before the neurorrhaphy) as this muscle repair can aid covering noble structures, including nerves and repaired vessels since the skin for closing the macroreimplant may be compromised.

With the advancement of techniques to prepare amputation stumps with target reinnervation of a muscle proximal to the amputation and techniques to sensitize cutaneous nerves with neurotization of the severed nerves of the stump (median, ulnar, and radial, according to availability and indication); the prognosis of patients without the possibility of reimplantation or with non-functional reimplanted limbs may improve with this evolution and new prostheses. ^(^ ^7^ However, studies describe even greater patient satisfaction and functional results, which may promote functional return and amputation stump sensitivity (including cases with unsatisfactory results), when compared to amputees and patients who received prostheses. ^(^ ^8^ ^), (^ ^9^ ^), (^ ^10^

In Brazil, GM/MS Ordinance 793^11^ establishes the care network for people with physical disabilities within the Unified Health System and provides for upper limb prostheses^12^ (including myoelectric devices following a multidisciplinary team’s analysis according to the steps to prepare and grant orthoses, prostheses, or auxiliary means of locomotion as per the World Health Organization). ^(^ ^13^

However, the adherence of patients with upper limb amputations to the available upper limb prostheses remains low. Reasons for their dissatisfaction include poor prosthesis function, low comfort, high prosthesis weight, and inadequate adjustment. ^(^ ^14^ ^)-(^ ^17^ Studies have shown^16^ ^), (^ ^18^ that patients who receive prosthetics soon after amputation, have more distal amputations, and receive adequate training, have greater long-term adherence to upper limb prosthesis. In our service, patients with traumatic wrist proximal amputations are often unable to undergo early prosthesis preparation following the WHO steps^13^ and have low prosthesis use adherence. Moreover, patients’ cultural preference for amputation usually configures a reimplantation attempt to the detriment of primary amputation. Studies have described that reimplanted patients suffer fewer psychological impacts, feel less disfigured, ^(^ ^19^ and have better function (including return to work) and greater satisfaction than patients who received prostheses regardless of functional outcomes. ^(^ ^15^ ^), (^ ^20^ Thus, our service prioritizes macroreimplantations whenever possible.

The Hand Surgery^21^ reference book indicates macroreimplantations for cold ischemic times ranging from six to 12 hours but states that from two to three hours of cold ischemia onward, amputation stumps begin to undergo muscle necrosis with risk of rhabdomyolysis and coagulopathy during macroreimplantation. On the other hand, Sabapathy et al. ^(^ ^22^ recommend no reimplantations for the middle-third forearm after seven hours of cold ischemia and from the distal third of the arm to the middle third of the forearm after six hours. Most cases in our tertiary referral service for complex cases of orthopedic trauma show that the time elapsed between the trauma and the beginning of the surgical procedure exceeds six hours of cold ischemia, averaging eight hours in our casuistry. Although our comparison between the mean time of ischemia of successful and unsuccessful macroreimplantation cases showed no statistical differences, the mean of the successful group (7.4 hours) was lower than the group with macroreimplantation loss (mean of 9.0 hours). This absence of statistical difference may stem from the number of treated cases. However, due to the rarity of this severe lesion with ischemia times equal to or above six hours, this sample is comparable with the literature.^23^ We believe that the ischemic time limit for macroreimplants should consider the severity of the injury; anesthetic and clinical teams’ technical and support conditions; and especially the adequate packaging of the amputated part, which arrives in inadequate preservation conditions in some cases.

The limitation of this study refers to its number of cases as this is a serious and rare accident in Brazil, but its strength lies in its consecutive inclusion of all cases with prolonged ischemia time, being one of the largest national series.

## CONCLUSION

Macroreimplants require immediate transport to specialized services. Moreover, temporary arterial catheterization to assist surgical management seems to fail to interfere with outcomes.
